# Ranibizumab Monotherapy or Combined with Laser versus Laser Monotherapy for Diabetic Macular Edema: A Meta-Analysis of Randomized Controlled Trials

**DOI:** 10.1371/journal.pone.0115797

**Published:** 2014-12-26

**Authors:** Guohai Chen, Wensheng Li, Radouil Tzekov, Fangzheng Jiang, Sihong Mao, Yuhua Tong

**Affiliations:** 1 Department of Ophthalmology, Quzhou People's Hospital, Quzhou, Zhejiang, PR China; 2 Xiamen Eye Center of Xiamen University, Xiamen, Fujian, PR China; 3 The Roskamp Institute, Sarasota, Florida, United States of America; 4 Department of Ophthalmology, University of South Florida, Tampa, Florida, United States of America; University of Utah (Salt Lake City), United States of America

## Abstract

**Objective:**

To evaluate the relative efficacy of ranibizumab (RBZ) monotherapy or combined with laser (RBZ + Laser) versus laser monotherapy for the treatment of diabetic macular edema (DME).

**Methods:**

A comprehensive literature search using PUBMED, ClinicalTrials.gov, and the Cochrane Library to identify randomized controlled trials (RCTs) comparing RBZ or RBZ + Laser to laser monotherapy in patients with DME. Efficacy estimates were determined by comparing weighted mean differences (WMD) in the change of best corrected visual acuity (BCVA) and central macular thickness (CMT) from baseline, and the risk ratios (RR) for the proportions of patients with at least 15 letters change from baseline. Safety analysis estimated the RR of cardiac disorders at 6 to 12 months in RBZ therapy vs. laser monotherapy. Statistical analysis was performed using the RevMan 5.1 software.

**Results:**

Seven RCTs were selected for this meta-analysis, including 1749 patients (394 patients in the RBZ group, 642 patients in the RBZ + Laser group, and 713 patients in the laser group). RBZ and RBZ + Laser were superior to laser monotherapy in the mean change of BCVA and CMT from baseline (WMD = 5.65, 95% confidence interval (CI), 4.44–6.87, P<0.00001; WMD  = 5.02, 95% CI, 3.83–6.20, P<0.00001, and WMD  = −57.91, 95% CI, −77.62 to −38.20, P<0.00001; WMD  = −56.63, 95% CI, −104.81 to −8.44, P = 0.02, respectively). The pooled RR comparing the proportions of patients with at least 15 letters improvement or deterioration were also in favor of RBZ and RBZ + Laser (RR = 2.94, 95% CI, 1.82–4.77, P<0.00001; RR = 2.04, 95% CI, 1.50–2.78, P<0.00001, and RR = 0.21, 95% CI, 0.06–0.71, P = 0.01; RR = 0.52, 95% CI, 0.29–0.95, P = 0.03, respectively). There were no significant differences between RBZ and RBZ + Laser for any of the parameters. There were no difference in the safety profile between RBZ and laser.

**Conclusion:**

RBZ and RBZ + Laser had better visual and anatomic outcomes than laser monotherapy in the treatment of DME. RBZ + Laser seemed to be equivalent to RBZ.

## Introduction

Diabetic retinopathy (DR) is the most frequent and severe ocular complication of diabetes mellitus, the leading cause of blindness in the working age population in developed countries [1]. Diabetic macular edema (DME) is a major contributor to vision loss and one of the main causes for decreased visual acuity in patients with DR [2]. The prevalence of DME increases from 0% to 3% in individuals recently diagnosed with diabetes to 28% to 29% in those with diabetes duration of over 20 years [3].

Focal/grid laser photocoagulation (laser), the standard of care for DME since 1985, was shown by the Early Treatment Diabetic Retinopathy Study (ETDRS) to reduce the risk for significant vision loss by 50%, but complete cessation of vision loss and/or improvements in visual acuity are rarely observed [4]. Based on the observation that vascular endothelial growth factor (VEGF) levels are increased in the retina and vitreous of eyes with DME [5], inhibiting VEGF may provide an alternative therapeutic approach for this condition. Recently, anti-VEGF agents have been reported to be efficacious in treating DME [6]–[8]. Among the anti-VEGF therapeutics, ranibizumab (RBZ, Lucentis, Genentech Inc., San Francisco, CA) is an antibody fragment with high binding affinity for VEGF-A, specifically developed for intraocular use [9]. Various studies have demonstrated that ranibizumab monotherapy is well-tolerated and significantly more effective than sham treatment in clinical trials for DME [8], [10].

Previous meta-analyses of clinical trials involving RBZ for DME have been focused mostly on safety issues, showing that RBZ for the treatment of DME did not increase the risk for thromboembolic events compared with other treatments, including laser, triamcinolone acetate or sham injection [11], [12]. Several systematic reviews on anti-VEGF agents for DME suggested that VEGF inhibitors are effective in treating DME, but in these cases RBZ was analyzed together with other VEGF inhibitors, including pegaptanib, aflibercept, and bevacizumab [13], [14]. To the best of our knowledge, only one study attempted a meta-analysis of randomized controlled trials (RCTs) comparing the efficacy of RBZ for DME, indicating that RBZ and RBZ combined with laser (RBZ + Laser) were more advantageous than non-drug treatment or laser monotherapy, and it included only three trials comparing RBZ or RBZ + Laser to laser monotherapy [15]. However, this meta-analysis was somewhat limited in scope, as no data were available to compare RBZ monotherapy and laser monotherapy and results from only one clinical trial were available to compare efficacy of RBZ vs. RBZ plus laser at 12 months. As more recent data are currently available, we decided to conduct an independent assessment of the available literature and to undertake a new meta-analysis of all available RCTs comparing the efficacy of RBZ or RBZ + Laser to laser monotherapy for the treatment of DME. In addition, a safety analysis was conducted for cardiovascular events for the period 6 to 12 months after initiation of therapy for the groups RBZ therapy (RBZ monotherapy and RBZ + Laser) vs. laser monotherapy.

## Materials and Methods

This meta-analysis was performed according to the PRISMA (Preferred Reporting Items for Systematic Review and Meta-Analyses) statement [16].

### Search Strategy

We conducted searches of PUBMED, ClinicalTrials.gov, and the Cochrane Library, using the terms *diabetic macular edema, DME, ranibizumab and lucentis*. A manual search was performed by checking the reference lists of original reports and review articles to identify studies not yet included in the computerized databases. The final search was carried out on June 2014, without restrictions regarding publication year or language.

### Inclusion and Exclusion Criteria

Articles were considered eligible for inclusion in the meta-analysis if the studies met the following inclusion criteria: 1. study design: RCT, 2. population: minimum age of 18 years with DME, 3. intervention: RBZ or RBZ + Laser versus laser monotherapy, and 4. outcome variables: at least one of the outcomes of interest discussed in the section Outcome Measures below. Abstracts from conferences, full texts without raw data available for retrieval, duplicate publications, letters, and review articles were excluded. When sequential reports on the same cohort of patients were considered, only the most recent report was included, and data that could not be obtained from this last publication were obtained from the previous reports.

### Outcome Measures

For efficacy, the primary outcome measures were the mean change in best corrected visual acuity (BCVA) from baseline and the mean change in central macular thickness (CMT) from baseline. The secondary outcome measures were the proportions of patients with change in BCVA by at least 15 letters improvement compared to baseline and by at least 15 letters deterioration compared to baseline.

For safety, the primary outcome measure was the relative risk of cardiovascular events including acute myocardial infarction, angina pectoris, cardiac failure chronic, myocardial infarction, myocardial ischemia etc. at 6 to 12 months after imitation of therapy.

### Data Extraction

The data were extracted independently by two reviewers (G.H.C. and W.S.L.). Disagreement was resolved by discussion. The information extracted from each study included the authors of each study, the year of reported, information on study design, location of the trial, duration of the study, number of subjects, the mean change in BCVA measured as ETDRS letters, the mean change in CMT measured with optical coherence tomography (µm), and the proportions of patients with a BCVA change of at least 15 letters improvement and 15 letters deterioration.

### Risk of Bias

Two review authors (F.Z.J. and S.H.M.) independently assessed the studies using the Cochrane Collaboration's “Risk of Bias” tool, which assesses sources of systematic bias according to the guidelines in chapter 8 of the Cochrane Handbook for Systematic Reviews of Intervention [17]. The criteria used for this were selection bias, performance and detection bias, attrition bias, and reporting bias. Studies were classed with high or unclear risk of bias for any of the first three components to be of low quality.

### Statistical Analysis

The quantitative data were entered into Cochrane Review Manager (RevMan, software version 5.1, Copenhagen, Denmark: The Nordic Cochrane Center, The Cochrane Collaboration, 2011). For continuous variables (e.g., BCVA), the weighted mean difference (WMD) was measured, while the risk ratios (RR) were measured for dichotomous variables (e.g., number of patients). Both outcomes were reported with a 95% confidence interval (CI). P<0.05 was considered statistically significant on the test for overall effect. The I^2^ statistic was calculated to assess heterogeneity between studies (P<0.05 was considered representative of significant statistical heterogeneity) [18]. If there was heterogeneity between studies, a random-effects model was applied to the data. Alternatively, a fixed effects model was used for pooling the data. Begg's rank correlation test and Egger's linear regression test were employed to quantitatively assess publication bias (P<0.05 was considered representative of significant statistical publication bias) [19], [20].

## Results

### Overall Characteristics of Selected Trials and Risk of Bias

**Figure 1:**
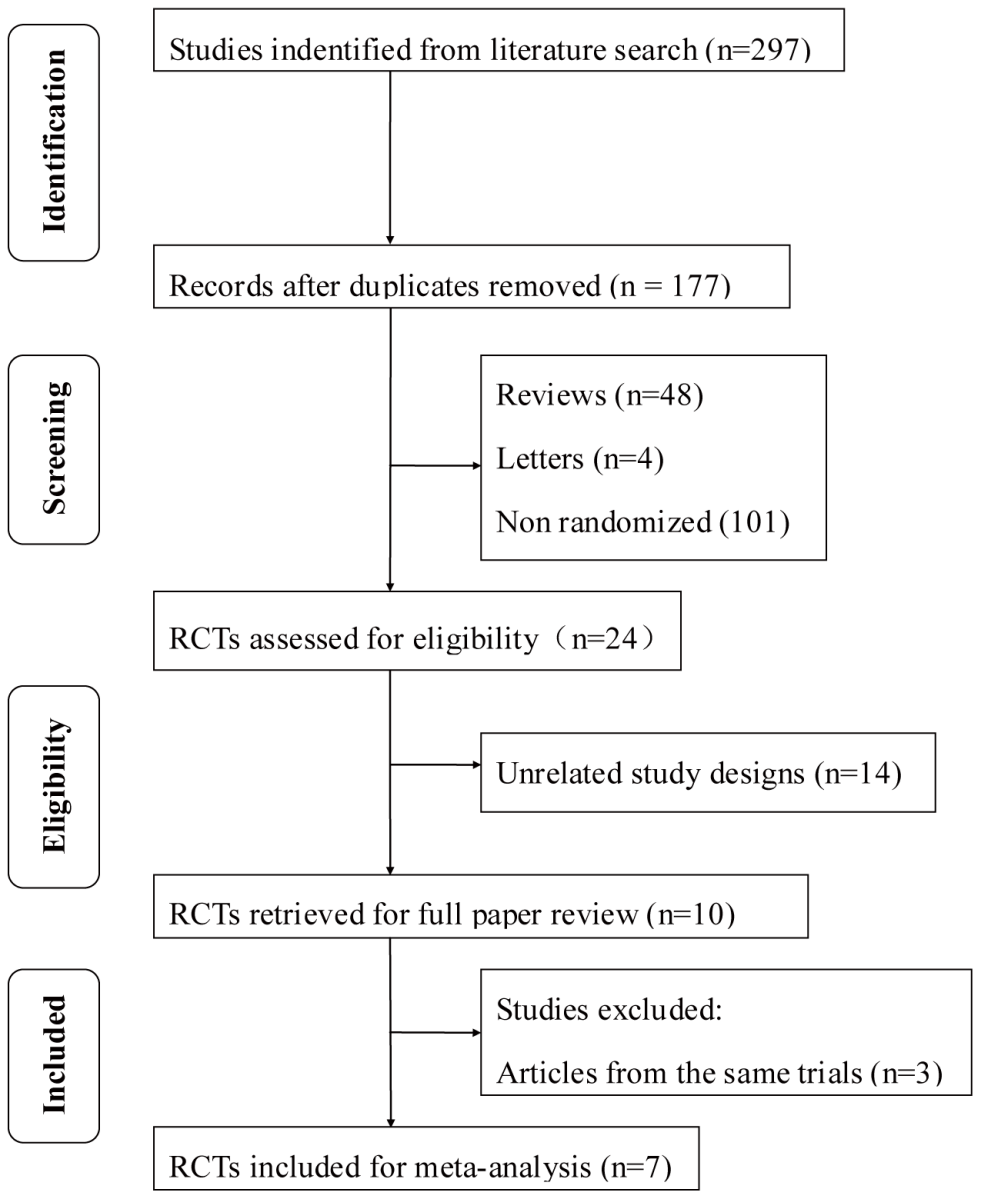
Flow diagram of studies included in this meta-analysis. RCT  =  randomized controlled trial.

A total of 297 articles were initially identified. Of these, 287 were rejected according to the exclusion criteria listed above. The 10 remaining articles with full texts that met the inclusion criteria were assessed [21]–[30]. For the three trials that were from the same cohort of patients, the most recent reports were selected [24], [26]. Hence, a total of seven studies were included in this meta-analysis. Fig. 1 provides a flow diagram of the search procedure and results. In total, there were 1749 patients included in the meta-analysis: 394 patients were included in the RBZ group, 642 patients in the RBZ + Laser group, and 713 patients in the laser monotherapy group. One trial (the LUCIDATE study) was a single center trial [30], while the others were all multicenter trials [21], [24], [26]–[29]. The characteristics of the studies included are summarized in Table 1. According to the quality assessment, just two of the seven studies [21], [30]were considered as low risk of bias (Table 2).

**Table 1:** Characteristics of Included Studies.

Study group (year)	Design	Location	Follow-up(mo)	Treatment	No. patients	Age (year)
DRCR.net (2011)	RCT	United States	24	0.5 mg RBZ + Laser	187	62
				Laser	293	63
LUCIDATE (2014)	RCT	United Kingdom	12	0.5 mg RBZ	22	64.9
				Laser	11	67.4
READ-2 (2013)	RCT	United States	36	0.5 mg RBZ	42	62
				0.5 mg RBZ + Laser	42	62
				Laser	42	62
RELATION (2012)	RCT	Germany	12	0.5 mg RBZ + Laser	85	63.5
				Laser	43	63.5
RESPOND (2013)	RCT	Canada	12	0.5 mg RBZ	81	62
				0.5 mg RBZ + Laser	78	62
				Laser	82	62
RESTORE (2011)	RCT	Europe, Turkey, Canada, and Australia	12	0.5 mg RBZ	116	62.9
				0.5 mg RBZ + Laser	118	64
				Laser	111	63.5
REVEAL (2012)	RCT	East Asia	12	0.5 mg RBZ	133	60.7
				0.5 mg RBZ + Laser	132	61.2
				Laser	131	61.5

RCT  =  randomized controlled trial, RBZ  =  ranibizumab, RBZ + Laser  =  ranibizumab combined with laser.

**Table 2:** Risk of Bias Assessment.

Study group (year)	Random sequence generation (selection bias)	Allocation concealment (selection bias)	Blinding of participants and personnel (performance bias)	Blinding of outcome assessment (detection bias	Incomplete outcome data (attrition bias)	Selective reporting (reporting bias)	Other bias
DRCR.net (2011)	U	U	L	U	L	L	L
LUCIDATE (2014)	L	L	L	L	L	L	L
READ-2 (2013)	U	U	U	U	L	L	L
RELATION (2012)	U	U	L	L	L	L	L
RESPOND (2013)	U	U	U	U	L	L	L
RESTORE (2011)	L	L	L	L	L	L	L
REVEAL (2012)	U	U	L	L	L	L	L

H, high risk; L, low risk; U, unclear risk.

### Best Corrected Visual Acuity

**Figure 2:**
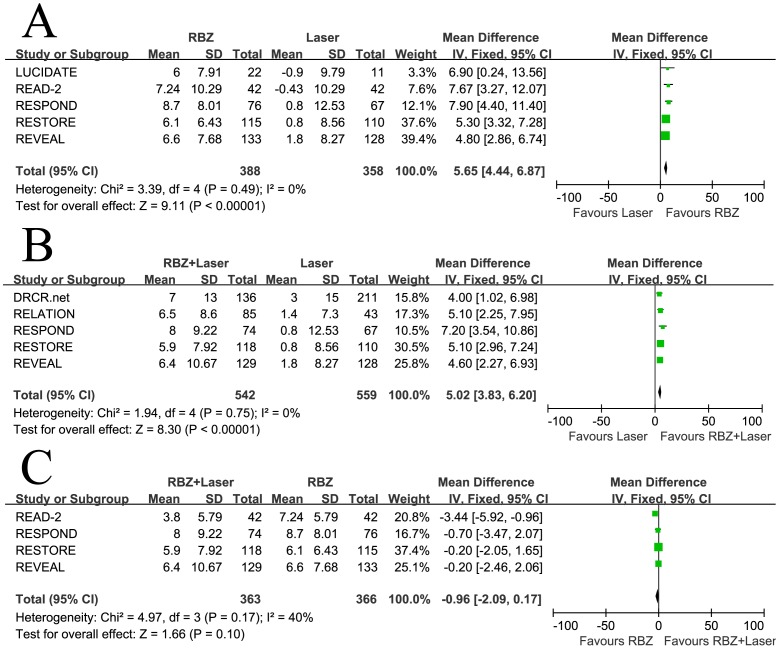
Mean change in best corrected visual acuity from baseline: (A) Ranibizumab versus laser, (B) Ranibizumab combined with laser versus laser, and (C) Ranibizumab combined with laser versus ranibizumab. RBZ  =  ranibizumab, RBZ + Laser  =  ranibizumab combined with laser; SD  =  standard deviation; IV  =  inverse variance; CI  =  confidence interval.

Five studies involving 746 patients compared RBZ to laser monotherapy in terms of the mean change in BCVA from baseline, five studies involving 1101 patients compared RBZ + Laser to laser monotherapy, and four studies involving 729 patients compared RBZ + Laser to RBZ. The combined results showed that administration of RBZ (either alone or in conjunction with laser) resulted in improvement of BCVA compared to application of laser treatment alone over 6 months. This superiority of RBZ and RBZ + Laser compared to laser treatment in terms of a positive effect on BCVA were highly significant (WMD  = 5.65, 95% CI, 4.44–6.87, P<0.00001 and WMD  = 5.02, 95% CI, 3.83–6.20, P<0.00001, respectively), with no heterogeneity identified (Fig. 2A and 2B). There was no significant difference between RBZ and RBZ + Laser arms in BCVA mean change from baseline (WMD  = −0.96, 95% CI, −2.09–0.17, P = 0.10), with no heterogeneity identified (Fig. 2C).

### Central Macular Thickness

**Figure 3:**
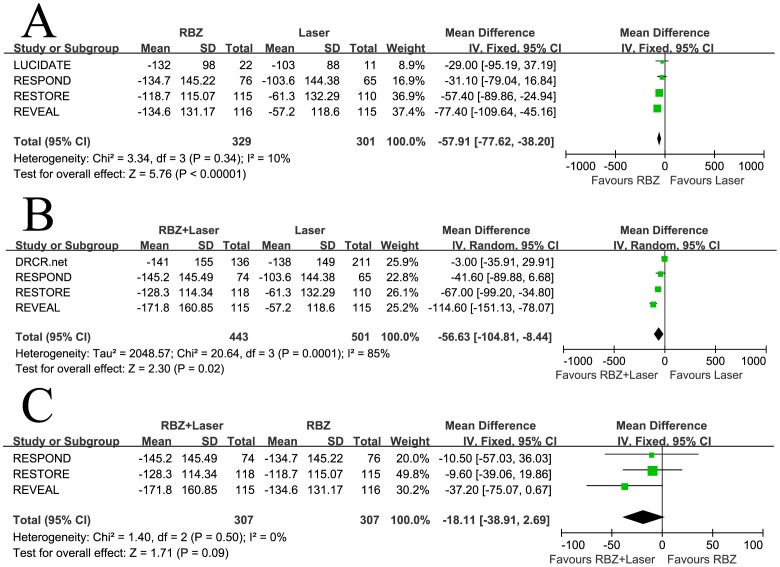
Mean change in central macular thickness from baseline: (A) Ranibizumab versus laser, (B) Ranibizumab combined with laser versus laser, and (C) Ranibizumab combined with laser versus ranibizumab. RBZ  =  ranibizumab, RBZ + Laser  =  ranibizumab combined with laser; SD  =  standard deviation; IV  =  inverse variance; CI  =  confidence interval.

Four studies involving 630 patients compared RBZ to laser monotherapy in terms of the mean change in CMT from baseline, four studies involving 944 patients compared RBZ + Laser to laser monotherapy, and three studies involving 614 patients compared RBZ + Laser to RBZ. The combined results showed that all the treatments were efficacious in reducing the CMT. RBZ and RBZ + Laser were both found to be more efficacious in reducing CMT when compared to laser monotherapy (WMD  = −57.91, 95% CI, −77.62 to −38.20, P<0.00001 and WMD  = −56.63, 95% CI, −104.81 to −8.44, P = 0.02), with no heterogeneity identified in the comparison of RBZ to laser monotherapy. However, heterogeneity was significant in the comparison of RBZ + Laser to laser monotherapy, and a random-effects model was applied to the data (Fig. 3A and 3B). When a comparison was made between RBZ + Laser to RBZ, the mean reduction of CMT from baseline was greater in the RBZ + Laser group, but this difference was not statistically significant (WMD  = −18.11, 95% CI, −38.91−2.69, P = 0.09), and no heterogeneity was identified (Fig. 3C).

### Secondary Outcomes

**Figure 4:**
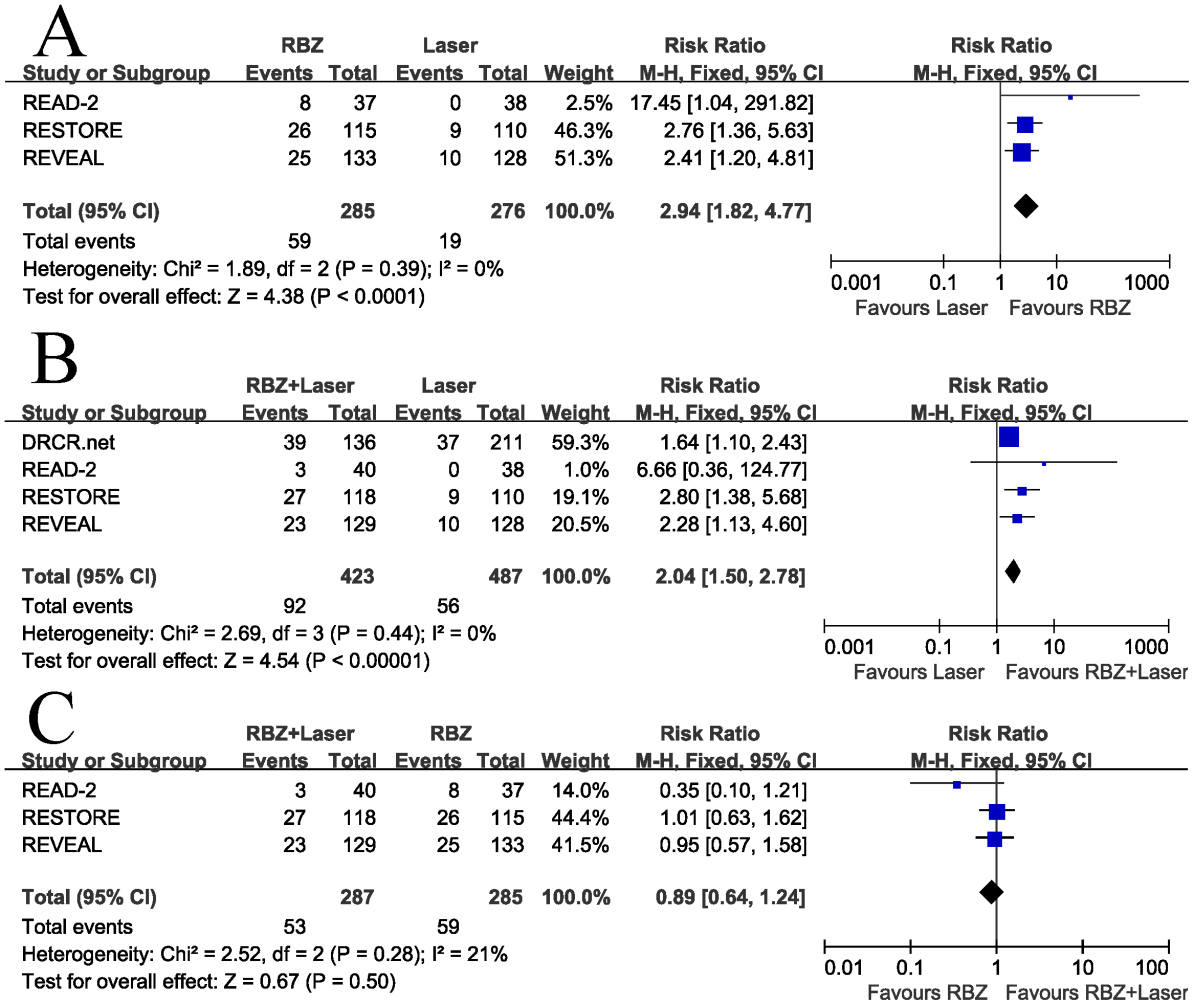
Gain of ≥15 letters from baseline: (A) Ranibizumab versus laser, (B) Ranibizumab combined with laser versus laser, and (C) Ranibizumab combined with laser versus ranibizumab. RBZ  =  ranibizumab, RBZ + Laser  =  ranibizumab combined with laser; M-H  =  Mantel-Haenszel statistics; CI  =  confidence interval.

**Figure 5:**
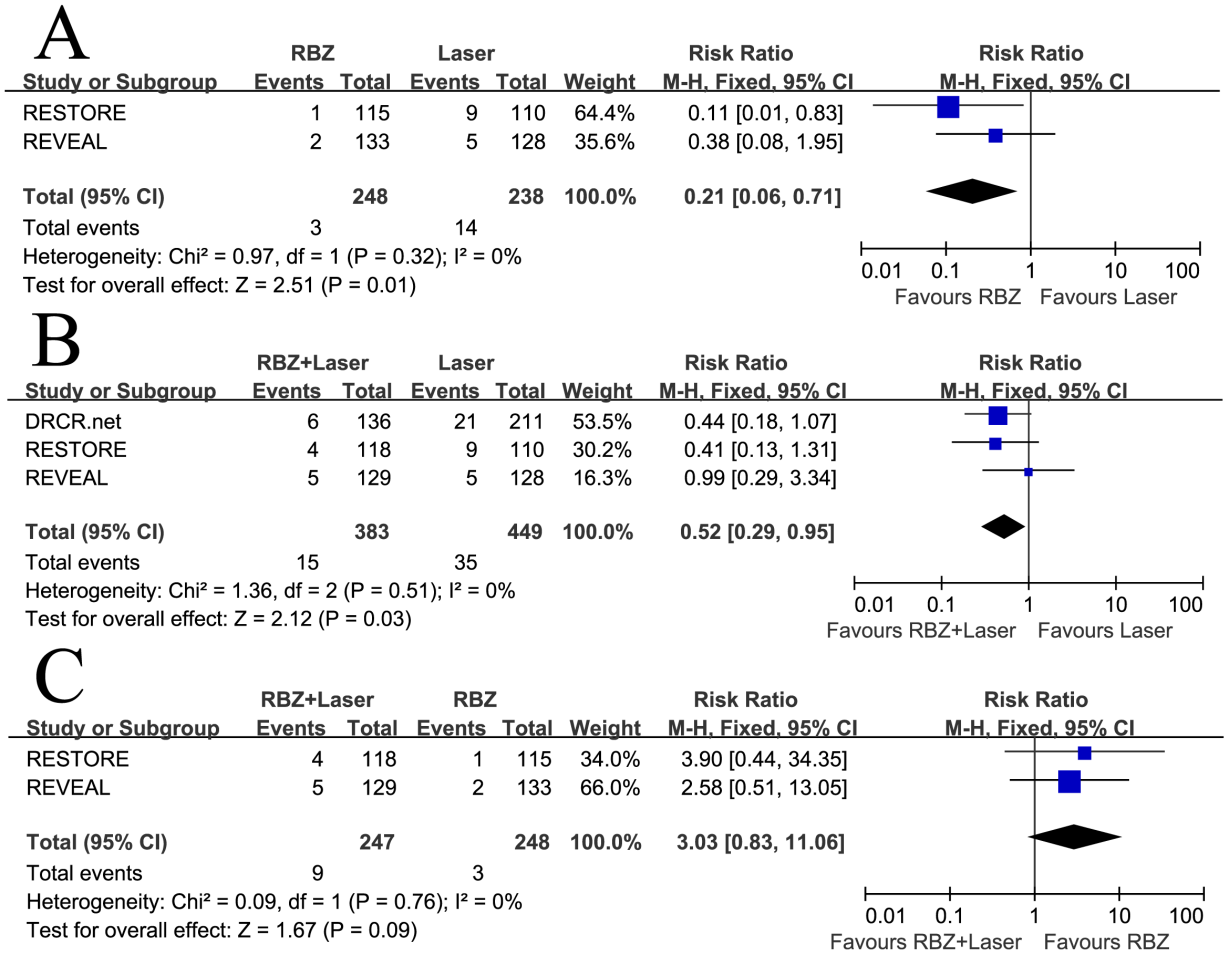
Loss of ≥15 letters from baseline: (A) Ranibizumab versus laser, (B) Ranibizumab combined with laser versus laser, and (C) Ranibizumab combined with laser versus ranibizumab. RBZ  =  ranibizumab, RBZ + Laser  =  ranibizumab combined with laser; M-H  =  Mantel-Haenszel statistics; CI  =  confidence interval.

The pooled RRs comparing the proportion of patients with at least 15 letters improvement were in favor of RBZ and RBZ + Laser arms (RR = 2.94, 95% CI, 1.82–4.77, P<0.00001 and RR = 2.04, 95% CI, 1.50–2.78, P<0.00001, respectively), with no heterogeneity identified (Fig. 4A and 4B). A significantly lower proportion of patients lost 15 letters or more in both the RBZ arms compared with the laser monotherapy (RR = 0.21, 95% CI, 0.06–0.71, P = 0.01 and RR = 0.52, 95% CI, 0.29–0.95, P = 0.03, respectively), with no heterogeneity identified (Fig. 5A and 5B). There was no significant difference between RBZ and RBZ + Laser arms with respect to the incidence of gain of 15 letters or more (RR = 0.89, 95% CI, 0.64–1.24, P = 0.50), with no heterogeneity identified (Fig. 4C). A lower proportion of patients with a loss of 15 letters or more was observed in RBZ compared with RBZ + Laser arm, but the difference was not statistically significant (RR = 3.03, 95% CI, 0.83–11.06, P = 0.09), with no heterogeneity identified (Fig. 5C). Begg's rank correlation test and Egger's linear regression test indicated no publication bias for any of the parameters.

### Cardiovascular Events

**Figure 6:**
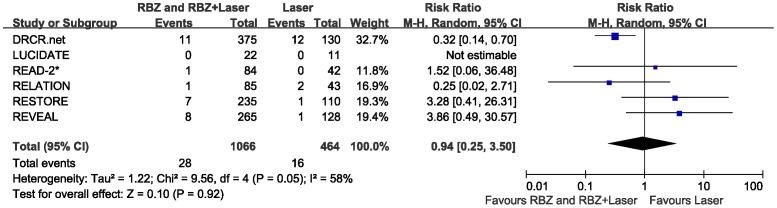
Cardiovascular adverse events for ranibizumab therapy (ranibizumab monotherapy and ranibizumab combined with laser) versus laser monotherapy for 6 to 12 months post initiation of therapy. RBZ  =  ranibizumab; RBZ + Laser  =  ranibizumab combined with laser; M-H  =  Mantel-Haenszel statistics; CI  =  confidence interval. *READ-2 study results at 6 months, all other studies at 12 months.

Six clinical trials were analyzed for reports of adverse cardiovascular events at 6–12 months post imitation of therapy. Overall, 28 events were reported in the RBZ therapy group (RBZ monotherapy and RBZ + Laser) (2.6%) and 16 events in the laser monotherapy group (3.4%). There was not statistically significant difference between the two groups in the RR for cardiovascular events (RR = 0.94, 95% CI 0.25–3.50, P = 0.92), with heterogeneity was identified and a random-effects model was applied to the data (Fig. 6).

## Discussion

Ranibizumab, as a recognized blocker of VEGF-A, has been found to significantly reduce total retinal thickness in DME [8], [10]. Therefore, theoretically, the application of RBZ alone or RBZ plus laser photocoagulation could enhance or even substitute the positive effects of the laser treatment monotherapy. Indeed, as our analysis demonstrates, RBZ + Laser demonstrated superior benefit in terms of improvement in both BCVA and CMT compared to laser monotherapy and the difference was statistically significant. In assessing the mean change in BCVA from baseline, both RBZ arms were superior to laser monotherapy in terms of BCVA improvement (RBZ vs. laser monotherapy: +5.6 letters and RBZ + Laser vs. laser monotherapy: +5.0 letters, respectively). Both RBZ arms were found to be more efficacious in reducing the abnormally increased central retinal thickness comparing to laser monotherapy (RBZ vs. laser monotherapy: 57.9 µm additional reduction in total retinal thickness and RBZ + Laser vs. laser monotherapy: 56.6 µm additional reduction, respectively). The mean change in CMT outcome suggested that laser treatment group may have higher incidence of chronic macular edema. Therefore, the laser treatment group may have a diminished potential for visual acuity improvement because of irreversible changes resulting from chronic macular edema. The RESTORE extension study found that the BCVA gained in the prior laser group was less than that observed in the prior RBZ groups after allowing RBZ in all groups during the extension study (5.4 letters in prior laser group, 6.7 letters in prior RBZ + Laser group, and 7.9 letters in prior RBZ group at month 24 and 6.0 letters in prior laser group, 6.7 letters in prior RBZ + Laser group, and 8.0 letters prior RBZ group at month 36) [31], [32]. Thus, overall, the results from the current study are the first meta-analysis data to support the superiority of RBZ + laser vs. laser monotherapy as a treatment option for DME up to 12 months after initiation of therapy.

No significant differences were observed with respect to improvements in BCVA between RBZ vs. RBZ + Laser treatment. The RBZ + Laser treatment paradigm had a tendency to be more efficacious in reducing CMT compared to RBZ (−148.67 µm vs. −128.67 µm), but the difference was not statistically significant. While a higher proportion of patients (3.64% vs. 1.21%) lost 15 letters or more in the RBZ + Laser group, this difference was not statistically significant. All studies included in this analysis applied the laser treatment at the initiation of RBZ application (within 1–10 days after the first RBZ injection). The presence of severe retinal edema at that time may make the laser treatment more difficult and less effective, due to the accumulation of subretinal fluid and thickening of the neural retina. This can lead to the absorption of more laser energy than usual and make the laser treatment less effective and result in additional visual loss. The 3-year DRCR.net results also suggest that laser treatment at the initiation of intravitreal RBZ is no better, and possibly even worse in terms of vision outcomes compared to RBZ plus deferring laser treatment for ≥24 weeks in eyes with DME [33].

The mean number of RBZ injections was analyzed in two studies included in this meta-analysis, showing that RBZ + Laser tended to reduce the need for continued injections of RBZ (6.8 versus 7.0 injections in the RESTORE study at one year and 3.3 versus 5.4 injections in the READ-2 study at three year) [21], [24]. Thus, to a certain extent, the combined therapy was thought to decrease the potential incidence of endophthalmitis and ease the economic burden on patients. In all other aspects, RBZ + Laser seemed to be equivalent to RBZ monotherapy. In summary, the current meta-analysis supports the option of using RBZ as a viable alternative in cases of DME.

Ranibizumab has been used in clinical settings for various indications for several years with an excellent safety record in terms of ocular adverse events. There have been some concern that prolonged administration of anti-VEGF agents can eventually affect systemic levels of VEGF and lead to increase in the incidence of undesirable systemic adverse events, including cardiovascular ones [34]. Thus, a recent study by Enders et al. demonstrated that in patients with AMD plasma levels of VEGF were decreased at 12 months post initiation of RBZ therapy by ∼39% [35] and it is likely that the same effect may be present in patients with DME. Our analysis of relative risk of cardiovascular events in the groups RBZ therapy (RBZ monotherapy and RBZ + Laser) vs. laser monotherapy at 6–12 months post imitation of therapy based on the combined results from six trials with a total enrollment of more than 1500 patients indicates no increased relative in the RBZ group compared laser monotherapy group. This confirms the good systemic safety record of RBZ up to 12 months post imitation of DME therapy and supports the strong safety profile of RBZ in other indications like nonvascular AMD, as presented in a recent meta-analysis [36].

This work may have some limitations. First, we cannot fully exclude publication bias. It is possible that some works, especially those published in languages other than English may have been missed. Although Begg's and Egger's tests demonstrated no evidence of publication bias, the results should be interpreted with caution. Second, a potential source of heterogeneity is different trial duration and lack of data reported in all phases of follow-up, but most studies had a 12-month follow-up time frame and all the conclusions from this meta-analysis apply mostly to this time period.

Current treatment options for DME allow for varied and increasingly complex combinations of treatment paradigms like laser monotherapy, combination of laser therapy with ant-VEGF agents (RBZ, bevacizumab, aflibercept), anti-VEGF monotherapy and sustained-release corticosteroid therapy (dexamethasone, either as a monotherapy or in combination with the other therapies). In this complex decision environment, the current meta-analysis provides additional support for some the choices like RBZ monotherapy and RBZ + laser therapy and confirms the cardiovascular safety of RBZ therapy up to 12 months.

## Supporting Information

S1 Checklist PRISMA Checklist.
(DOC)
